# Fibronectin in Fracture Healing: Biological Mechanisms and Regenerative Avenues

**DOI:** 10.3389/fbioe.2021.663357

**Published:** 2021-04-16

**Authors:** Jonathan Klavert, Bram C. J. van der Eerden

**Affiliations:** Department of Internal Medicine, Erasmus University Medical Center, Rotterdam, Netherlands

**Keywords:** fibronectin, fracture healing, biomaterial, extracellular matrix, bone, regenerative medicine, tissue engineering, angiogenesis

## Abstract

The importance of extracellular matrix (ECM) proteins in mediating bone fracture repair is evident, and fibronectin (FN) has emerged as a pivotal regulator of this process. FN is an evolutionarily conserved glycoprotein found in all tissues of the body, and functions in several stages of fracture healing. FN acts as a three-dimensional scaffold immediately following trauma, guiding the assembly of additional ECM components. Furthermore, FN regulates cellular behavior via integrin-binding and growth factor-binding domains, promoting downstream responses including cell recruitment, proliferation and differentiation. Due to its diverse functions, the development of FN-based strategies to promote fracture healing is under intense research. In this review, we discuss the recent advancements in utilizing FN-based biomaterials, showing promise in tissue engineering and regenerative medicine applications.

## Introduction

The skeletal system is involved in many roles, including mechanical load bearing and movement, soft tissue protection, and as a supportive niche for hematopoietic cells (Calvi et al., 2003). The performed functions of bone are dependent on its structure, thus resolving structural defects are crucial for maintaining homeostasis (reviewed in Florencio-Silva et al., 2015). Although bone is a highly dynamic tissue, large bone defects still represent a clinical challenge. Due to being one of the most common injuries worldwide (Amin et al., 2014), bone fractures cause high morbidity and economic burden to society. The elderly in particular are at an increased risk for developing fractures due to the high prevalence of osteoporosis in this demographic, a disease characterized by brittle bones estimated to affect >14 million people in the United States alone (National Osteoporosis Foundation, 2002).

Approximately 5–10% of bone fractures result in delayed healing or non-union, which may require surgical intervention (Einhorn, 1998). The currently accepted gold standard for treatment in such cases remains the use of bone graft, but are not without limitations (reviewed by Faour et al., 2011). Donor morbidity and limited tissue availability are concerns using autologous bone grafts, and while concern of sufficient tissue amounts can be bypassed with allogenic material, risk of disease transmission remains an issue (Delloye et al., 2007).

## Bone Extracellular Matrix

These concerns and limitations highlight the need for developing novel strategies aimed at promoting bone regeneration, which can be achieved by further elucidating the molecular mechanisms involved in fracture healing. Of particular interest is the role of extracellular matrix (ECM), which comprises approximately 90% of bone tissue in mass (v/v) (Mansour et al., 2017). Additionally, the ECM is known to be a pivotal regulator of cellular behaviors including adhesion, migration, proliferation, and differentiation (Becerra-Bayona et al., 2012; Zeitouni et al., 2012). The significance of ECM function in bone regeneration has been demonstrated by the improved (clinical) performance of decellularized allogenic grafts, showing similar success to autogenic grafts (Al-Abedalla et al., 2015).

Many ECM molecules have been studied extensively throughout the years. This review seeks to integrate the findings related to one particular ECM protein, namely fibronectin (FN), which seems to be functionally relevant across several stages of fracture healing.

## Stages of Fracture Healing

Fracture healing is a tightly controlled process, which after the injury causing trauma can be divided into four main stages: (1) inflammation, (2) soft callus formation, (3) hard callus formation, and (4) bone remodeling. There seems to be temporal overlap between aforementioned stages of fracture healing (Schindeler et al., 2008), but making such distinctions is nevertheless practical to describe the general events that take place.

**FIGURE 1:**
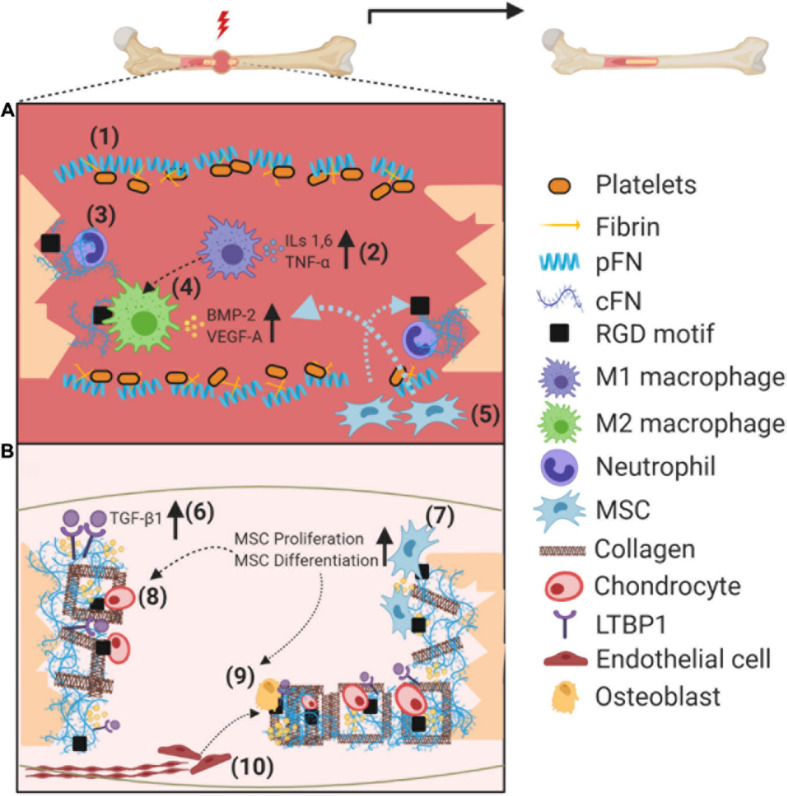
Schematic illustration on the role of fibronectin (pFN and cFN) during fracture healing. **(A)** Soon after the fracture, pFN from the bloodstream in association with fibrin promotes clotting near the fracture site to form a hematoma **(1)**. Acute inflammation is initiated by macrophages (M1), recruiting further immune cells via cytokine secretion **(2)**. Neutrophils lay the groundwork for healing by secreting cFN as an “emergency ECM” **(3)**. Cell binding to cFN RGD motifs switch macrophages to an anti-inflammatory (M2) phenotype, which secrete growth factors to recruit, among others, MSCs and ECs **(4)**. Recruited MSCs infiltrate injury site and dock on integrin binding sites **(5)**. **(B)** cFN orchestrates the deposition of additional proteins such as LTBP1 and collagen to the ECM, increasing availability of TGF-β ligands, along with entrapment of additional growth factors such as BMP-2 **(6)**. MSCs bound to cFN increase their proliferation rate via integrin signaling, followed by differentiation toward chondro/osteoprogenitors **(7)**. Docking to cFN and growth factor regulation promotes cartilage formation via chondrocytes to stabilize the fracture **(8)**. Attachment and proliferation of osteoblasts is promoted by integrin signaling, forming the hard callous **(9)**, coupled to the recruitment/proliferation of endothelial cells and establishment of new vasculature also in part via integrin signaling **(10)**. Abbreviations used: plasma fibronectin (pFN), cellular fibronectin (cFN), mesenchymal stem cell (MSC), latent TGF-binding protein 1 (LTBP1), transforming growth factor β (TGF-β).

The initial stage of inflammation arises from damage to soft tissue surrounding the fracture, which leads to bleeding that later develops into a hematoma. Inflammation itself is tightly regulated as the inflammatory cascade is essential to initiate fracture healing, but must be appropriately resolved at later stages (for a recent review, see Maruyama et al., 2020). Pro-inflammatory cytokines secreted by resident macrophages promotes the recruitment of additional immune cells, including neutrophils and monocytes, in a positive feedback loop of acute inflammation (reviewed in Baht et al., 2018). Such an inflammatory microenvironment is crucial in recruiting mesenchymal stem cells (MSCs) to the site of injury via chemotaxis (i.e., interleukins [ILs] 1 and 6, TNF-alpha; Figure 1A). MSCs have the ability to differentiate into cells which regenerate skeletal elements (Buravkova and Anokhina, 2008; Schindeler et al., 2008; Watson et al., 2014), and this process is promoted by cellular crosstalk with immune cells (Chang et al., 2008; Raggatt et al., 2014; Vi et al., 2015). Conversely, MSCs have been described to possess an immunomodulatory role, mediated by release of soluble factors and cell–cell interactions, thus resolving the acute inflammatory response (reviewed in Munir et al., 2018).

Most fractures follow the endochondral ossification pathway, in which the final resulting bone is preceded by a soft callus composed of cartilage (Gerstenfeld et al., 2003). This soft callus serves to provide mechanical stability to the fracture, and acts as a rudimentary template for bone formation (Schindeler et al., 2008). To establish vasculature, endothelial cells (ECs) then invade the soft callous; a process in which MSCs play a crucial role, by producing angiogenic factors including vascular endothelial growth factor-A (VEGF-A) and platelet-derived growth factor-BB (PDGF-BB) known to enhance migration and proliferation of ECs (Chen et al., 2008). Recent studies have demonstrated that angiogenesis and osteogenesis are tightly coupled processes positively regulating each other, with intimate crosstalk occurring between specific EC subsets and bone progenitor cells (Kusumbe et al., 2014; Ramasamy et al., 2014).

Hard callous formation occurs in stable areas surrounding the soft callous, as the new bone forms in association with new blood vessels. Osteoblasts secrete the osteoid (primarily composed of type I collagen, osteopontin, and osteocalcin) into the ECM, which are then mineralized to form hydroxyapatite crystals (Li et al., 2014). Remodeling of the mineralized (hard) callous is the final stage of fracture healing in which specialized cells–namely osteoclasts–resorb mineralized bone resulting in surface gaps then re-filled by osteoblasts (Schindeler et al., 2008).

## Fibronectin Structure and Assembly

**FIGURE 2:**
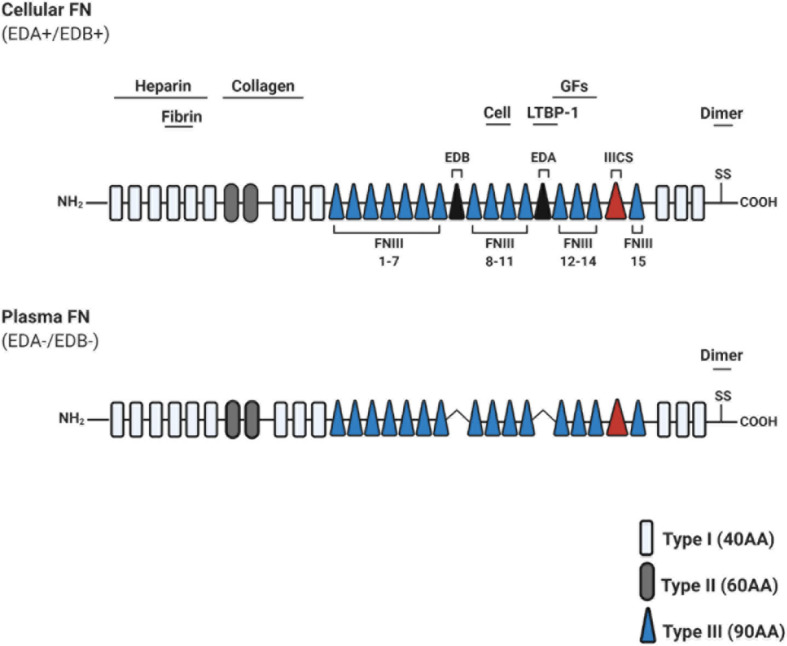
Schematic illustration of fibronectin primary structure. Fibronectin (FN) is constitutively composed of evolutionarily conserved repeats: Type I (FNI; 12 units), Type II (FNII; 2 units), and Type III (FNIII; 15 units). Two FN monomers bind at the C-terminal dimerization site. Cellular FN isoforms are composed of alternatively spliced extra domains A/B (EDA/EDB), and variable portions of type III connecting segment (IIICS). Plasma FN lacks both EDA and EDB domains entirely. Known heparin, collagen, and fibrin-binding domains are depicted throughout FN, along with a promiscuous growth factor (GF)-binding domain (FNIII repeats 12–14). FNIII repeats 9–10 contain RGD motifs to bind integrins which mediate cell attachment and downstream signaling. Abbreviations used: growth factors (GFs), amino acids (AA), extra domain A/B (EDA/EDB), type III connecting segment (IIICS), arginine-glycine-aspartate (RGD).

Fibronectin is an evolutionarily conserved glycoprotein, existing as a dimer composed of two nearly identical ∼250 kDa subunits (Pankov and Yamada, 2002; Figure 2). FN is a ubiquitous component of ECM in all tissues, which regulates adhesion, growth, and differentiation of cells (Yamada and Clark, 1996). FN possesses key functions in dynamic tissue remodeling throughout embryonic development (Davidson et al., 2004; Larsen et al., 2006), and has been linked to disease progression in multiple contexts (Oyama et al., 1989; Inufusa et al., 1995; Wan et al., 2013).

Fibronectin protein can exist in 20 isoforms, arising from alternative splicing of a single pre-mRNA molecule (Kosmehl et al., 1996). Structurally, FN consists of types I, II, and III repeating units (FNI-FNIII) and a C-terminal dimerization site. FN domains include binding sites for ECM molecules (i.e., collagen, heparin, fibrin, and other FN molecules), and for cell-binding via integrin receptors (for a recent review on FN structure, see Bradshaw and Smith, 2014; Figure 2). FN is secreted in both a soluble form, termed plasma FN (pFN), and a less soluble form termed cellular FN (cFN). cFN protein structure is distinguished from pFN by the inclusive splicing of extra domain(s) A and/or B (EDA+/EDB+), promoted in part via SF2/ASF or SRp40 splicing enhancers, respectively (reviewed in White et al., 2008; Figure 2).

Fibronectin matrix structure is assembled into higher order fibrils of >10 nm in diameter and tens of micrometers in length (Larsen et al., 2006). Three-dimensional assembly of FN is a highly dynamic cell dependent process, and this cell-matrix crosstalk is guided by key physical parameters (for a comprehensive review on FN assembly, see Singh et al., 2010 Annu Rev). Cell contractile forces are essential for FN matrix formation (Halliday and Tomasek, 1995), as cells tether FN–FN interactions. Conversely, contraction of the FN matrix exposes additional (“cryptic”) binding sites for additional FN molecules (Aguirre et al., 1994; Hocking et al., 1994). FN conformational alterations have also been demonstrated to affect cellular responses via integrin-binding specificity (Keselowsky et al., 2003, 2005) and growth factor (GF) availability (Wan et al., 2013).

## Fibronectin in Fracture Healing

Immediately following injury, pFN in the bloodstream promotes clotting near injured vessels, and seems to require fibrin association to enhance platelet aggregation (Wang et al., 2014). pFN has also been proposed to limit excessive clot formation distal from the injury site *in vivo* (Wang and Ni, 2016). Although the described mechanism of pFN has not been studied in bone fracture models, it is reasonable to speculate that similar processes are involved during initial stages of blood vessel disruption and subsequent hematoma formation (Figure 1A). Unlike pFN, which is exclusively synthesized by hepatocytes, cFN is synthesized by several different cell types including fibroblasts, ECs, chondrocytes, and osteoblasts (Mao and Schwarzbauer, 2005). There is transcriptomic and immunohistochemical evidence that cFN is expressed, and dynamically regulated, in several stages of fracture healing (Liu et al., 2005; Kilian et al., 2008).

### Integration With ECM Through Integrins

Cellular FN is assembled into a complex fibrillar matrix in a cell dependent process, and this cell-matrix interaction is largely mediated by integrins (Mao and Schwarzbauer, 2005). cFN binding to integrin receptors depend on a particular amino acid motif, consisting of Arginine-Glycine-Aspartate (RGD), which provide anchoring points for cells to the ECM. Activated integrin receptors can, in part, direct cytoskeletal arrangements, which stretch the FN molecules and promote a positive feedback loop of fibril formation (Antia et al., 2008). Integrin α5ß1 expressed on osteogenic cells and fibroblasts, along with αVß3 expressed on osteogenic cells and ECs, seem to play a crucial role in mediating cellular attachment to bone ECM (Parisi et al., 2019b).

In addition to regulating cell adhesion and fibril formation, FN-mediated integrin signaling can also alter cellular behavior itself. For instance, recombinant FN fragment containing the integrin-binding domain (FNIII 9–10) was shown to promote proliferation and differentiation of osteoblasts *in vitro* (Kim et al., 2003). Comparable performance of this FN fragment to native FN protein suggests that integrin signaling is a main factor in osteoblast proliferation and differentiation, thus positively contributing to fracture healing. The FNIII 9–10 integrin-binding domain was also found to enhance migration of both MSCs and ECs *in vitro*, providing further evidence of its importance in bone regeneration (Martino et al., 2011). FN-mediated integrin signaling can also alter cellular phenotypes, as has been observed for macrophages. Lv et al. (2018) have recently demonstrated that macrophages interact with FN via ß1 integrins, converting (pro-inflammatory) M1 macrophages into an anti-inflammatory M2 phenotype *in vitro* (Figure 1A).

There is evidence that cFN is synthesized by inflammatory cells, neutrophils in particular, which infiltrate the fracture hematoma immediately within 48 h post-injury (Bastian et al., 2016). It is hypothesized that cFN acts as an “emergency ECM” at this timepoint, before stromal cells (i.e., MSCs and their progeny) infiltrate approximately 5 days post-injury and further modify the matrix (Bastian et al., 2016). This is consistent with the structure of cFN, which contains binding domains for various ECM proteins such as collagens I and III, fibulin-1, fibrinogen, thrombospondin-1, and others (reviewed in Dallas et al., 2005). Such an “emergency ECM” composed of cFN could act as an initial scaffold for additional ECM molecules. Several studies have demonstrated that the three-dimensional cFN matrix plays an important role in orchestrating the spatiotemporal deposition of additional ECM molecules previously mentioned (Sottile and Hocking, 2002; Sottile et al., 2007).

### Interaction With Growth Factors

Fibronectin has also been shown to bind a variety of GFs relevant to bone regeneration (Martino et al., 2011). A study conducted by Martino and Hubbell (2010) using recombinant FN fragments which strongly bind such GFs, significantly enhanced bone regeneration in rat models of critical-sized bone defect (Martino et al., 2011). In particular, the authors report that FN type III repeats 9–10 and 12–14 (FN III 9–10/12–14) promoted GF binding for chemotactic recruitment and proliferation of ECs and MSCs (i.e., VEGF-A, BMP-2, and PDGF-BB; Figure 1B; Martino et al., 2011). Binding of GFs to FN III 9–10/12–14 is unlikely to be the only mechanism involved, as functional synergy with integrins (namely α5ß1 and αVß3) was evident.

Other crucial GFs in fracture healing are those of the transforming growth factor (TGF) family, which have been shown to recruit, among others, MSCs, fibroblasts and chondro/osteoprogenitors (Narine et al., 2006; Mendelson et al., 2011). There is a body of evidence supporting a non-redundant role of cFN in sequestering TGF ligands into the ECM. The cFN scaffold orchestrates deposition of latent TGF-binding protein 1 (LTBP-1), which seems to interact preferentially with the EDA+ domain of cFN, resulting in subsequent TGF-beta ligand incorporation (Klingberg et al., 2018; Figure 1B, blue circles). In addition to its role in chemotactic recruitment, TGF-β has been shown to promote callous formation via chondrocyte and osteoblast proliferation (Li et al., 2005), and differentiation (Bonewald and Dallas, 1994; Zhang et al., 2021). Interestingly, TGF-β administration also strongly upregulates VEGF mRNA in osteoblasts *in vitro*, which could promote osteogenesis via regulating the coupled process of angiogenesis (Saadeh et al., 1999). pFN is also know to bind GFs, as it contains the main GF-binding domains (FN III 12–14) (Prasad and Clark, 2018). Coupled with the observations that pFN from the circulation contributes to local tissue ECM, its potential role in promoting bone healing via GF binding cannot be ruled out (Moretti et al., 2007).

## Recent Advancements in Fibronectin-Based Biomaterials

Due to its diverse properties in regulating both osteogenic and supporting cell types, along with orchestrating the assembly of various other ECM proteins and GFs, incorporating FN into regenerative medicine applications is under intensive research (Xing et al., 2017; Cheng et al., 2018; Escoda-Francolí et al., 2018; Guillem-Marti et al., 2018; Parisi et al., 2019a; Sánchez-Garcés et al., 2020; Toffoli et al., 2020; Trujillo et al., 2020; Zhang et al., 2020).

Lee et al. (2019) recently developed an FN fusion protein, containing FNIII 9–10 domain and elastin like peptides (FN-ELP) to more reliably recapitulate mechanical properties of native ECM. FN-ELP significantly enhanced MSC adhesion, proliferation, and osteogenic differentiation *in vitro*. This approach could thus provide improved *ex vivo* expansion and differentiation of MSCs into desired cell type(s) for stem cell-based therapies. Another noteworthy finding demonstrated that the GF binding domain (FNIII 12–14) could also facilitate cell attachment and spreading to titanium surface when an RGD gain-of-function mutation was introduced within (Guillem-Marti et al., 2019).

In addition to FN fragments which can be immobilized, the development of hydrogels also seems to be a promising avenue. Such hydrogels can potentially be administered surrounding the fracture site directly, which could alleviate the issue of difficult-to-access areas when using grafts. Ao et al. (2020) recently developed a hydrogel composed of FN, fibrin glue, and heparin. In addition to reliably mimicking native ECM, this hydrogel could sustain the slow release of BMP-2–circumventing an issue persistent in previous carriers of BMP-2 administration. Their hydrogel managed to efficiently induce osteoblastic differentiation of MC3T3-E1 cells *in vitro*, and significantly promote fracture healing *in vivo* (Ao et al., 2020). Another promising biomaterial seems to be the use of FN in combination with nanotubules–mimicking collagen fibers–which is both injectable and can self-assemble (Zhou et al., 2020). The authors report significantly improved human MSC adhesion and migration into the “Nano-Matrix” *in vitro* (Zhou et al., 2020).

## Future Outlook

Despite the progress made, several biological questions remain unanswered. The negative relationship between aging and fracture healing has been well-described, and aberrations in FN functionality could mediate this reduced healing capacity. Interestingly, it has been shown that there is a significant effect of age on alternative splicing of FN both *in vivo* and *in vitro* (Magnuson et al., 1991), but the functional consequences and whether it is biologically significant in fracture healing, is still unknown. A connection between age-related FN (alternative) splicing and TGF-β signaling could potentially be made. As LTBP1 preferentially interacts with EDA+ FN (Klingberg et al., 2018), and levels of EDA+ FN are subject to age related changes, the deposition of LTBP1 could likely be affected.

Interestingly, relatively little is known about the role of FN during bone remodeling. There have been some demonstrations which suggest FN can upregulate osteoclast activity *in vitro* (Gramoun et al., 2010), but studying the complete mechanisms underlying this could prove fruitful for our knowledge on bone remodeling. Furthermore, although studies have conclusively demonstrated the importance of immune cell crosstalk in promoting bone regeneration (Chang et al., 2008; Raggatt et al., 2014; Vi et al., 2015), it remains unexplored in the context of FN-mediated fracture healing.

## Concluding Remarks

Bone fractures will become increasingly common in the aging population, causing significant morbidity and financial burden. The involvement of ECM in fracture healing has been well-described along with its key molecular players, but recent evidence is further accumulating for the crucial role of FN. As it plays an important role in various stages of the healing cascade, studying FN functionality further, along with its incorporation into treatment strategies, could be of great utility.

Much attention has been devoted to developing FN-based biomaterials, and such three-dimensional scaffolds–especially those which can exert temporal control on GF availability–seem highly promising. Further *in vivo* studies are warranted to evaluate their efficacy, perhaps investigating not just their effect on osteoprogenitors and MSCs, but to the highly relevant ECs and immune cell subsets as well. Several of the research lines summarized in this review have also highlighted the functional importance in utilizing recombinant FN fragments. Such recombinant fragments could pave the way for upscaling this approach, making it more feasible to translate into treatment strategies.

## Author Contributions

JK performed the literature research, wrote the manuscript, and designed the figure. BE revised the manuscript. Both authors contributed to the article and approved the submitted version.

## Conflict of Interest

The authors declare that the research was conducted in the absence of any commercial or financial relationships that could be construed as a potential conflict of interest.
